# Sodium levels and immunotherapy efficacy in mRCC patients with bone metastases: sub analysis of Meet-Uro 15 study

**DOI:** 10.3389/fimmu.2024.1361010

**Published:** 2024-07-05

**Authors:** Martina Catalano, Sara Elena Rebuzzi, Marco Maruzzo, Ugo De Giorgi, Sebastiano Buti, Luca Galli, Giuseppe Fornarini, Paolo Andrea Zucali, Melanie Claps, Silvia Chiellino, Ilaria Zampiva, Stefania Pipitone, Riccardo Ricotta, Mariella Sorarù, Veronica Mollica, Marianna Tudini, Lucia Fratino, Veronica Prati, Orazio Caffo, Francesco Atzori, Franco Morelli, Giuseppe Prati, Franco Nolè, Francesca Vignani, Alessia Cavo, Marilena Di Napoli, Andrea Malgeri, Emanuele Naglieri, Alessio Signori, Giuseppe Luigi Banna, Pasquale Rescigno, Linda Cerbone, Lorenzo Antonuzzo, Giandomenico Roviello

**Affiliations:** ^1^ Department of Health Sciences, Section of Clinical Pharmacology and Oncology, University of Firenze, Firenze, Italy; ^2^ Medical Oncology Unit, Ospedale San Paolo, Savona, Italy; ^3^ Department of Internal Medicine and Medical Specialties (Di.M.I.), University of Genoa, Genoa, Italy; ^4^ Oncology 1 Unit, Department of Oncology, Istituto Oncologico Veneto (IOV) - Istituti di Ricovero e Cura a Carattere Scientifico (IRCCS), Padova, Italy; ^5^ Department of Medical Oncology, IRCCS Istituto Romagnolo per lo Studio dei Tumori (IRST) "Dino Amadori", Meldola, Italy; ^6^ Department of Medicine and Surgery, University of Parma, Parma, Italy; ^7^ Medical Oncology Unit, University Hospital of Parma, Parma, Italy; ^8^ Medical Oncology Unit 2, Azienda Ospedaliero-Universitaria Pisana, Pisa, Italy; ^9^ Medical Oncology Unit 1, IRCCS Ospedale Policlinico San Martino of Genova, Genova, Italy; ^10^ Department of Biomedical Sciences, Humanitas University, Pieve Emanuele, Milan, Italy; ^11^ Department of Oncology, IRCCS Humanitas Research Hospital, Rozzano, Milan, Italy; ^12^ SS Oncologia Medica Genitourinaria, Fondazione IRCCS Istituto Nazionale dei Tumori, Milano, Italy; ^13^ Medical Oncology Unit, IRCCS Policlinico San Matteo, Pavia, Italy; ^14^ Section of Innovation Biomedicine-Oncology Area, Department of Engineering for Innovation Medicine (DIMI), University of Verona, Verona, Italy; ^15^ Medical Oncology Unit, Department of Oncology and Hemathology, University Hospital of Modena, Modena, Italy; ^16^ Oncology Unit, IRCCS MultiMedica, Sesto san Giovanni, Milan, Italy; ^17^ UOC Oncologia, AULSS 6 Euganea, Ospedale di Camposampiero, Padova, Italy; ^18^ Medical Oncology, IRCCS Azienda Ospedaliero-Universitaria di Bologna, Bologna, Italy; ^19^ Medical Oncology, St. Salvatore Hospital, L’Aquila, Italy; ^20^ Department of Medical Oncology, Centro di Riferimento Oncologico di Aviano CRO-IRCCS, Aviano, Italy; ^21^ Oncology Unit, Michele e Pietro Ferrero Hospital, Azienda Sanitaria Locale (ASL) CN 2, Verduno, Italy; ^22^ Department of Medical Oncology, Santa Chiara Hospital, Trento, Italy; ^23^ SSD Oncologia Medica, Azienda Sanitaria Locale (ASL) Sulcis, Cagliari, Italy; ^24^ Medical Oncology Department, Casa Sollievo Della Sofferenza Hospital, IRCCS, San Giovanni Rotondo, Italy; ^25^ Department of Oncology and Advanced Technologies AUSL - IRCCS Reggio Emilia, Reggio Emilia, Italy; ^26^ Medical Oncology Division of Urogenital & Head & Neck Tumors, IEO, European Institute of Oncology IRCCS, Milan, Italy; ^27^ Division of Medical Oncology, Mauriziano Hospital, Turin, Piemont, Italy; ^28^ Oncology Unit, Villa Scassi Hospital, Genova, Italy; ^29^ Department of Urology and Gynecology, Istituto Nazionale Tumori IRCCS Fondazione G. Pascale, Naples, Italy; ^30^ Department of Medical Oncology, Fondazione Policlinico Campus Bio-Medico, Roma, Italy; ^31^ Division of Medical Oncology, IRCCS Istituto Tumori “Giovanni Paolo II”, Bari, Italy; ^32^ Department of Health Sciences, Section of Biostatistics, University of Genova, Genoa, Italy; ^33^ Faculty of Science and Health, School of Pharmacy and Biomedical Sciences, Portsmouth Hospitals University NHS Trust, University of Portsmouth, Portsmouth, United Kingdom; ^34^ Candiolo Cancer Institute, FPO-IRCCS, Candiolo, Turin, Italy; ^35^ Department of Medical Oncology, San Camillo Forlanini Hospital, Rome, Italy; ^36^ Clinical Oncology Unit, Careggi University Hospital, Florence, Italy; ^37^ Department of Experimental and Clinical Medicine, University of Florence, Florence, Italy

**Keywords:** renal cell carcinoma, bone metastases, immunotherapy, sodium levels, efficacy outcomes

## Abstract

**Background:**

Immune-checkpoint inhibitors (ICIs) have significantly improved metastatic renal cell carcinoma (mRCC) prognosis, although their efficacy in patients with bone metastases (BMs) remains poorly understood. We investigated the prognostic role of natremia in pretreated RCC patients with BMs receiving immunotherapy.

**Materials and methods:**

This retrospective multicenter study included RCC patients with BMs receiving nivolumab as second-line therapy or beyond. Inclusion criteria involved baseline sodium levels (pre-ICI) and sodium levels after 4 weeks of nivolumab initiation (post-ICI). The population was divided into two groups based on the median value, and response rates, progression-free survival (PFS), and overall survival (OS) were assessed.

**Results:**

Among 120 eligible patients, those with pre-treatment sodium levels ≥140 mEq/L showed longer OS (18.7 *vs.* 12.0 months, p=0.04). Pre-treatment sodium levels ≥140 mEq/L were associated with better OS compared to levels <140 mE/L (18.7 *vs.* 12.0, p=0.04). Post-treatment sodium levels ≥140 mEq/L were associated with improved PFS (9.6 *vs*. 3.2 months) and OS (25.1 *vs.* 8.8 months) (p=0.05 and p<0.01, respectively). Patients with consistent sodium levels ≥140 mEq/L at both time points exhibited the best outcomes compared to those with lower values (PFS 11.5 *vs.* 3.3 months and OS 42.2 *vs.* 9.0 months, respectively, p<0.01). Disease control rate was significantly higher in the latter group (p<0.01). Multivariate analysis confirmed the prognostic significance of sodium levels.

**Conclusion:**

Elevated sodium levels (≥140 mEq/L) pre- and post-ICI treatment correlate with better survival outcomes in mRCC patients with BMs. This finding suggests sodium level assessment as a potential prognostic factor in these patients and warrants further investigation, particularly in combination immunotherapy settings.

## Introduction

Checkpoint inhibitor therapy is a form of cancer immunotherapy targeting Cytotoxic T-Lymphocyte Antigen 4 (CTLA4), programmed cell death-1 (PD-1), and programmed cell death-ligand 1 (PD-L1) to restore immune system function (1). Immune-checkpoint inhibitors (ICI)-based combinations, such as pembrolizumab/axitinib, nivolumab/cabozantinib, pembrolizumab/lenvatinib and nivolumab/ipilimumab, are now the standard of care for first-line metastatic renal cell carcinoma (mRCC) (2–4). These combinations have shown particular efficacy among intermediate- and poor-risk patients based on the International Metastatic RCC Database Consortium (IMDC). Following first-line treatment with vascular endothelium growth factor receptor-tyrosine kinase inhibitor (VEGFR-TKI), nivolumab, a PD-1 blocking antibody, is a recognized options for second-line therapy (5, 6). However, it is important to acknowledge that not all mRCC patients experience long-term benefits from ICIs, whether administered as monotherapy or in combination (7, 8).

RCC is a heterogeneous disease characterized by a highly variable clinical course, spanning from indolent to rapidly progressive disease (9, 10). Notably, one-third of RCC patients present with bone metastases (BMs) at diagnosis, which is strongly associated with a worse prognosis (11) and a median overall survival (OS) that ranges from 12 to 28 months (12).

While ICIs have demonstrated substantial efficacy against visceral disease, their effectiveness in patients with bone metastases remains insufficiently explored. Data indicates that BMs are associated with inferior progression-free survival (PFS) and OS compared to other metastatic sites during ICI treatment (13), although, additional investigations are imperative.

Consequently, identifying biomarkers to distinguish patients who are most likely to benefit from ICI from those who are not represents an unmet clinical need in practice and research. While potential biomarkers such as molecular and genomic signatures are currently under investigation, none have achieved validation for daily clinical use.

Hyponatremia, defined as a serum sodium level below 135 mEq/L, serves as an independent prognostic factor in various solid malignancies, including RCC (14–17). It has been linked to poorer prognosis and shorter cancer-specific survival in mRCC patients treated with different drug classes (18, 19). Recently, lower, but in range, sodium levels have been correlated with worse prognosis in mRCC patients receiving TKIs or nivolumab therapy (20, 21). Preclinical investigations are currently exploring the influence of sodium levels on cancer progression and the modulation of immune responses, with the potential to unveil novel concurrent therapeutic strategies in the next future (22–24).

This multicenter retrospective analysis was conducted to evaluate the impact of sodium levels on response rates and survival outcomes in RCC patients with BMs receiving nivolumab as second-line therapy or beyond.

## Materials and methods

### Patients and treatment

We conducted a retrospective analysis of clinical data pertaining to consecutive patients diagnosed with RCC with BMs who underwent treatment with nivolumab as second-line therapy or beyond (after one or more TKI lines). This analysis encompassed the period from October 2015 to November 2019 and involved thirty Oncology Centers in Italy (subgroup analysis of Meet-URO 15 study) (25). The criteria for inclusion in this study entailed the availability of serum sodium measurements at two distinct time points: baseline (mentioned as pre-ICI) and approximately four weeks after the initial administration (mentioned as post-ICI). We systematically documented various demographic and clinical parameters for all patients, including histological subtype, risk classification based on IMDC criteria, Karnofsky-Performance Status (PS) (26), neutrophil-to-lymphocyte ratio (NLR), the choice of first-line therapy, and serum sodium levels.

Nivolumab was initially administered intravenously at a dose of 3 mg/kg administered every two weeks. Subsequently, in May 2018, a fixed dose of 240 mg every two weeks or 480 mg every four weeks was employed, based on local clinical practices, and continued until either disease progression or the onset of unacceptable toxicity. Ethical clearance for this study was granted by the Ethics Regional Ethical Committee of Liguria, under registration number 068/2019. The written informed consent was obtained from all patients.

### Treatment evaluation

Serum sodium levels were routinely examined as part of laboratory assessments: at baseline and before the initiation of each subsequent therapy cycle. Normal natremia was defined as a serum sodium level within the range of ≥135 and ≤145 mEq/L, as per the laboratory’s established reference values. Treatment response was conducted at three-month intervals using computed tomography scans, and assessed following the Response Evaluation Criteria in Solid Tumor (RECIST) version 1.1 (27). Treatment efficacy was appraised in terms of both OS and PFS. Any adverse events (AEs) occurring during nivolumab administration were strictly monitored by the investigators and promptly reported in accordance with the Common Terminology Criteria of Adverse Events (CTCAE) version 5.0 (28). A comprehensive analysis was undertaken to explore potential prognostic correlations, encompassing variables such as age, gender, histological subtype, history of previous surgeries, IMDC score, performance status, number of metastatic sites, and levels of serum sodium both before and after the start of systemic treatment.

### Efficacy outcomes

The aim of this investigation was to examine the correlation between sodium levels (either before or after ICI treatment) and the treatment effectiveness and survival outcomes of RCC patients diagnosed with BMs and treated with nivolumab as second-line therapy or beyond. To achieve this objective, patients were separated into two cohorts based on the median value of their serum sodium concentrations. The primary endpoints were PFS, which was defined as the duration from the start of treatment to either disease progression or death, and OS, defined as the interval between the initiation of treatment and death from any cause. In addition, secondary outcome measures encompassed disease control rate (DCR), defined as the proportion of patients attaining complete response (CR), partial response (PR), or stable disease (SD), as well as the objective response rate (ORR), indicating the proportion of patients achieving CR or PR (27).

### Statistical analysis

Continuous variables were represented by medians, accompanied by ranges indicating the lowest and highest values, while categorical variables were expressed as numerical counts and corresponding percentages. The estimation of PFS and OS employed the Kaplan-Meier method, with group comparisons executed through the log-rank test. Hazard ratios (HRs) and their associated two-sided 95% confidence intervals (CI) were computed using the Cox proportional hazard model.

Prospective prognostic factors for PFS and OS were evaluated in the initial univariate analysis, with variables exhibiting a p-value of ≤0.05 being chosen for inclusion in the subsequent multivariate analysis. The multivariate analysis was adjusted to account for potential confounding variables (*i.e*., including the IMDC, Karnofsky-PS, score, NLR, prior nephrectomy, and pre- and post-ICI serum sodium levels).

To examine secondary outcomes, the variables were dichotomized, and Fisher’s exact test was employed to establish correlations between dichotomized serum sodium values and clinical and biochemical parameters. The sample size for our study was deliberately determined to achieve a power of 0.80, ensuring a statistically meaningful capacity to detect significant effects or associations. The statistical analyses were carried out using STATA version 9.

## Results

### Patient characteristics

A total of 120 patients met the eligibility criteria and were included in the study. Their baseline characteristics are summarized in the **Table 1**. The median age of the patients was 76 years, with a range from 44 to 84 years. Eighty-seven (72.5%) were male, 108 (90.2%) had clear cell histology, and 104 (86.7%) were categorized as intermediate-poor risk according to the IMDC criteria. Nearly all patients (79.2%) had a Karnofsky-PS of 80% or higher (patients able to carry on normal activity and to work without it special care needed). Visceral metastases and lymph node involvement were observed in the 83.3 and 55.8% of patients, respectively. Sunitinib, pazopanib, or alternative therapeutic options represented the first line treatments received by 60.8%, 36.7% and 2.5% of patients, respectively. Nivolumab was administered as a second-, third, or further-line treatment in 70.3%, 21.6% and 7.5% of patients, respectively. Nephrectomy was previously performed in 81.7% of the cases. The serum sodium levels ranged from 129 to 147 mEq/L, with a median value of 140 mEq/L.

**Table 1:** Patients’ baseline characteristics.

	All patients (N=120)
**Age** Median (range)	76 (44-84)
**Gender, **n (%) Male Female	87 (72.5) 36 (27.5)
**Histology, **n (%) Clear-cell RCC **Papillary-RCC** **Chromophobe-RCC** Xp11Sarcomatoid	108 (90.2) 6 (5) 4 (3.2) 1 (0.8) 1 (0.8)
**Previous nephrectomy n (%)** Yes	98 (81.7)
**Karnofsky performance status, **n (%) ≥80%	95 (79.2)
**IMDC score, **n (%) Intermediate-poor	104 (86.7)
**Sites of metastases, **n (%) Lymph-nodal Visceral	67 (55.8) 100 (83.3)
**First-Line Therapy, **n (%) Sunitinib Pazopanib Other	73 (60.8) 44 (36.7) 3 (2.5)
**Nivolumab line, **n (%) Second line Third line ≧̸Fourth line	85 (70.3) 26 (21.6) 9 (7.5)
**Pre-treatment Na+** (mEq/L) Median (range)	140 (129-147)

RCC, renal cell carcinoma; IMDC, international metastatic renal cell carcinoma database consortium; TKI, tyrosine kinase inhibitor.

Additional details regarding the baseline characteristics of patients categorized based on their median serum sodium levels (<140 or ≥140 mEq/L) at both pre- and post-ICI assessments are reported in the **Supplementary Files** (**Supplementary Tables S1**-**S3**). In the pre-treatment evaluation, 69 patients (57.5%) had a serum sodium level ≥140 mEq/L, while 51 patients (42.5%) had a level <140 mEq/L.

At the post-ICI evaluation, 56 patients (46.6%) showed a serum sodium level ≥140 mEq/L, while 64 patients (53.3%) had a level <140 mEq/L.

Notably, no statistically significant differences were observed in terms of demographic and clinical features between patients with serum sodium <140 mEq/L and those with levels ≥140 mEq/L in the pre-ICI evaluation.

### Efficacy outcomes and best responses

The evaluation of efficacy outcomes and the best response was established on median serum sodium levels (140 mEq/L) and the timing of assessment as shown in **Table 2**. At the time of data cut-off, November 2023, the median follow-up was 22.1 months with a mean survival time of 15.5 months (95% CI 9.9-20.3).

**Table 2:** Best response, PFS and OS according to serum sodium values.

	ORR n (%)	DCR n (%)	Median PFS months (95% CI)	Median OS months (95% CI)
**All patients** (N=120)	26 (21.7)	59 (49.2)	4.7 (2.5-6.8)	15.5 (10.1-21.0)
**Pre-treatment Na+, (n)** ≥140 mEq/L (69) <140 mEq/L (51)	13 (18.8) 13 (25.5) p=0.5	36 (52.2) 23 (45.1) p=0.5	7.9 (2.0-13.8) 4.1 (2.7-5.5) p=0.18	18.7 (10.5-27.3) 12.0 (5.0-18.9) p=0.04
**Post-trearment Na+, (n)** ≥140 mEq/L (56) <140 mEq/L (64)	13 (23.2) 13 (20.3) p=0.8	35 (62.5) 24 (37.5) P<0.01	9.6 (6.17-13.0) 3.2 (2.5-3.9) p=0.05	25.10 (14.2-36.0) 8.8 (3.3-14.3) p<0.01
**Pre-and post- Na+, (n)** ≥140 mEq/L (41) <140 mEq/L (79)	10 (24.4) 16 (20.2) p=0.6	29 (70.4) 30 (38.0) p<0.01	11.5 (4.4-18.5) 3.3 (2.6-4.0) p<0.01	42.6 (16.8-68.3) 9.0 (4.64-13.35) p<0.01

ORR, overall response rate; DCR, disease control rate; PFS, progression free survival; OS, overall survival; Na+, serum sodium; CI, confidence interval.

**Figure 1:**
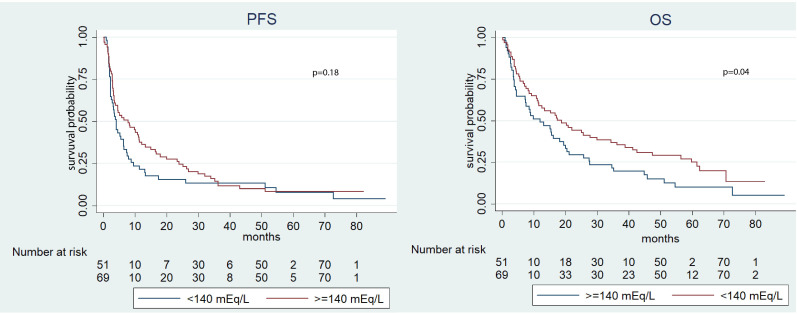
Kaplan-Meier survival estimate according to pre-ICI sodium value.

**Figure 2:**
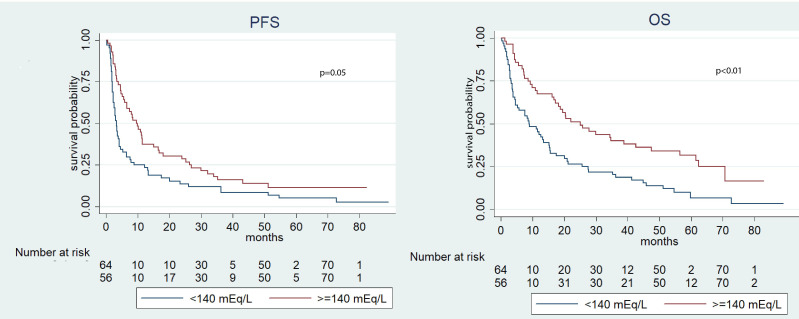
Kaplan-Meier survival estimate according to post-ICI sodium value.

**Figure 3:**
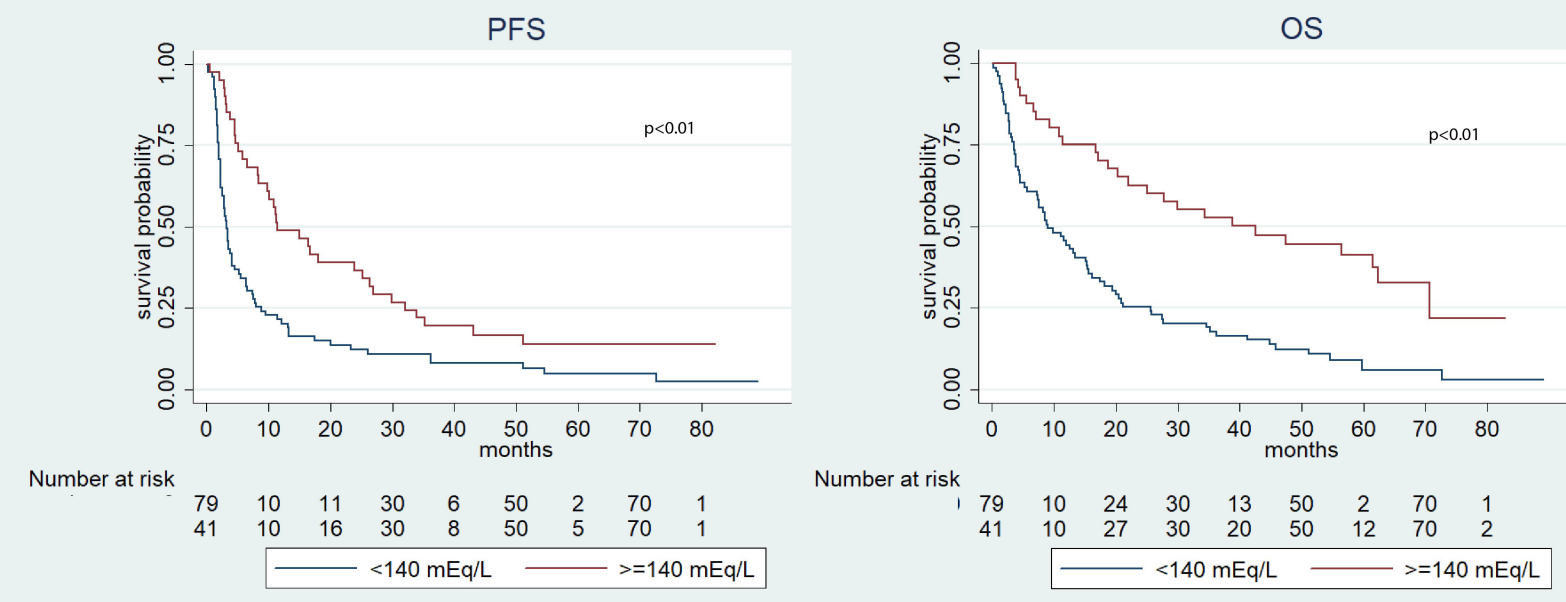
Kaplan-Meier survival estimate according to both pre-and post-ICI sodium serum values.

Significantly, the median OS was longer in the group characterized by higher pre-treatment sodium levels (≥140 mEq/L) if compared to the cohort with lower levels (<140 mEq/L) (18.7 *vs.* 12.0 months, p=0.04). In contrast, no significant disparities were observed in terms of PFS (p=0.18) (**Figure 1**). During the post-ICI assessment, patients with serum sodium levels ≥140 mEq/L showed prolonged median PFS and OS compared to those with levels <140 mEq/L (p=0.05 and p<0.01, respectively) (**Figure 2**). This pattern was consistently observed in patients with natremia ≥140 mEq/L at both pre- and post-ICI assessments when compared to those with at least one natremia level <140 mEq/L (p<0.01 and p<0.01) (**Figure 3**).

No differences were identified in the ORR between patients with serum sodium levels above or below 140 mEq/L at the pre-ICI assessment (p=0.50), post-ICI assessment (p=0.80), or both pre- and post-ICI evaluations (p=0.60). Nevertheless, patients with serum sodium levels ≥140 mEq/L at the post-ICI evaluation and those with levels ≥140 mEq/L at both pre- and post-ICI evaluations exhibited an improved DCR compared to patients with lower levels (p=0.01 and p<0.01, respectively).

In the univariate survival analysis, several factors were found to be associated with PFS, including prior nephrectomy (HR 0.59, 95% CI 0.36-0.95; p=0.03), Karnofsky-PS ≥80% (HR 0.44, 95% CI 0.28-0.71, p<0.01), IMDC intermediate-poor risk score (HR 1.77, 95% CI 1.24-2.52, p<0.01), NLR (HR 1.53, 95% CI 1.15-2.05, p<0.01), post-ICI serum sodium ≥140 mEq/L (HR 0.58, 95% CI 0.40-0.85, p<0.01), and serum sodium ≥140 mEq/L at both pre- and post-ICI evaluations (HR 0.48, 95% CI 0.32-0.72, p<0.01). For OS, significant associations were observed with the following factors: prior surgery (HR 0.48, 95% CI 0.29-0.81, p<0.01), Karnofsky-PS ≥80% (HR 0.38, 95% CI 0.23-0.61, p<0.01), IMDC intermediate-poor risk score (HR 2.76, 95% CI 1.37-5.55, p<0.01), NLR (HR 1.76, 95% CI 1.28-2.41, p<0.01), pre-ICI serum sodium ≥140 mEq/L (HR 0.66, 95% CI 0.44-0.98, p=0.04), post-ICI serum sodium ≥140 mEq/L (HR 0.49, 95% CI 0.32-0.73, p<0.01), and serum sodium ≥140 mEq/L at both pre- and post-ICI evaluations (HR 0.36, 95% CI 0.23-0.57, p<0.01).

In the multivariate analysis, these variables retained their statistical significance concerning both PFS and OS, except for the IMDC score, which did not demonstrate a significant association with PFS. Additionally, previous nephrectomy did not demonstrate a significant association with either PFS or OS. For detailed results of the univariate and multivariate analyses, please refer to **Tables 3** and **4**.

**Table 3:** Univariate analysis for PFS and OS.

	HR	CI 95%	*p*
Progression free Survival			
**Age** >75	0.89	0.55-1.43	0.6
**Gender** Male	0.67	0.44-1.02	0.1
**Histology** Clear-cell RCC	1.80	0,90-3.58	0.1
**Previous nephrectomy** Yes	**0.59**	**0.36-0.95**	**0.03**
**Karnofsky performance status** ≥80%	**0.44**	**0.28-0.71**	**<0.01**
**IMDC score** Intermediate-poor	**1.77**	**1.24-2.52**	**<0.01**
**NLR** (≥3.2 vs <3.2)	**1.53**	**1.15-2.05**	**<0.01**
**Lymph-nodal metastases** Yes	0.74	0.51-1.01	0.1
**Visceral metastases** Yes	1.12	0.68-1.76	0.6
**First-Line Therapy** Sunitinib vs pazopanib	1.00	0.68-1.48	0.9
**Nivolumab line** Second vs ≥ third	1.45	0.95-2.21	0.1
**Pre-treatment Na+** Na+ ≥140 mEq/L	0.77	0.53-1.13	0.2
**Post-treatment Na+** Na+ ≥140 mEq/L	**0.58**	**0.40-0.85**	**<0.01**
**Pre-and post- Na+** Na+ ≥140 mEq/L	**0.48**	**0.32-0.72**	**<0.01**
Overall Survival			
**Age** >75	0.98	0.59-1.62	0.9
**Gender** Male	0.73	0.47-1.13	0.1
**Histology** Clear-cell RCC	1.78	0.86-3.68	0.1
**Previous nephrectomy** Yes	**0.48**	**0.29-0.81**	**<0.01**
**Karnofsky performance status** ≥80%	**0.38**	**0.23-0.61**	**<0.01**
**IMDC score** Intermediate-poor	**2.76**	**1.37-5.55**	**<0.01**
**NLR** (≥3.2 vs <3.2)	**1.76**	**1.28-2.41**	**<0.01**
**Lymph-nodal metastases** Yes	0.88	0.59-1.31	0.5
**Visceral metastases** Yes	1.05	0.61-1.80	0.9
**First-Line Therapy** Sunitinib vs pazopanib	0.94	0.62-1.41	0.8
**Nivolumab line** Second vs ≥ third	1.07	0.69-1.65	0.7
**Pre-treatment Na+** Na+ ≥140 mEq/L	**0.66**	**0.44-0.98**	**0.04**
**Post-treatment Na+** Na+ ≥140 mEq/L	**0.49**	**0.32-0.73**	**<0.01**
**Pre-and post- Na+** Na+ ≥140 mEq/L	**0.36**	**0.23-0.57**	**<0.01**

RCC, renal cell carcinoma; IMDC, international metastatic renal cell carcinoma database consortium; TKI, tyrosine kinase inhibitor; HR, hazard ratio; Na, sodium; CI, confidence interval; P, p value.

Bold values is related to the significance of p value.

**Table 4:** Multivariate analysis for PFS and OS.

	HR	CI 95%	p	HR	CI 95%	*p*	HR	CI 95%	*p*
	Progression free Survival			Overall Survival			Overall Survival		
**Previous nephrectomy** Yes	0.80	0.54-1.39	0.4	0.91	0.76-1.45	0.2	0.81	0.51-1.6589	0.3
**Karnofsky performance status** ≥80%	**0.54**	**0.34-0.84**	**<0.01**	**0.6575**	**0.53-0.89**	**0.03**	**0.70**	**0.54-0.92**	**0.03**
**IMDC score** Intermediate-poor	1.78	0.89-3.44	0.1	**2.50**	**1.45-5.81**	**<0.01**	**3.35**	**1.99-5.80**	**<0.01**
**NLR** (≧̸3.2 vs <3.2)	**1.45**	**1.08-1.90**	**0.02**	**1.80**	**1.44-2.90**	**<0.01**	**1.77**	**1.41-2.80**	**<0.01**
**Pre-treatment Na+** Na+ ≥140 mEq/L	**_**	**_**	**_**	**0.90**	**0.70-0.98**	**0.04**	**_**	**_**	**_**
**Post-treatment Na+** Na+ ≥140 mEq/L	**0.61**	**0.45-0.88**	**<0.01**	**_**	**_**	**_**	**0.65**	**0.41-0.85**	**<0.01**

RCC, renal cell carcinoma; PFS, progression-free survival; OS, overall survival; IMDC, international metastatic renal cell carcinoma database consortium; HR, hazard ratio; Na, sodium; CI confidence interval; P, p value.

Bold values is related to the significance of p value.

## Discussion

The treatment of mRCC has seen significant advancements, particularly in the use of initial immunotherapy, which has contributed to improve patient prognosis (29). Nivolumab remains the standard of care as a second-line therapy after demonstrating superiority over everolimus in terms of response rate, PFS, OS, and quality of life in the pivotal CheckMate 025 trial (6). However, a significant portion of patients does not respond adequately to ICI therapy or experiences limited benefits. Prognostic markers for immunotherapy response identified in other tumor types have not been replicated in mRCC. The Meet-URO 15 study introduced a more accurate prognostic score incorporating clinical factors (bone metastasis) and inflammatory indices (NLR), outperforming IMDC alone (25). The presence of BMs was identified as an independent prognostic factor for lower OS. However, the study also identified a subgroup of patients with BMs (IMDC favorable-risk category and low NLR) associated with a very positive prognosis, indicating the need to consider bone metastasis prognostics within the patient’s clinical and immunological context.

Approximately one-third of mRCC patients have BMs, with median OS ranging from 12 to 28 months (12). While ICIs have shown efficacy against visceral disease, their effectiveness in patients with BMs remains insufficiently explored. The predictive and prognostic role of BM remains unclear, possibly influenced by niches and pathological bone loss hindering immune activation, affecting memory T and B lymphocytes, and cytotoxic T cell production. T-regs may play a role in balancing osteoclastic and osteoblastic activity, with potential immunosuppressive effects in the bone microenvironment (30). Limited data suggests that BMs may be associated with inferior PFS and OS during ICI treatment compared to other metastatic sites (13). Conversely, in an exploratory *post hoc* analysis of CheckMate 9ER study indica ted that patients with BMs experienced tumor regression with nivolumab plus cabozantinib compared to sunitinib, as did patients with other metastatic sites (31). Furthemore, subgroup analyses of the Checkmate 025 study showed a major improvement in ORR in patients with BMs treated with nivolumab compared to everolimus (26% *vs.* 6%, respectively) (32). However, the potential benefits of ICIs on BMs require further investigation.

Our study represents the first attempt to evaluate the prognostic significance of sodium levels in mRCC patients with BMs receiving nivolumab as a ≥2nd line therapy. We found that a pre-ICI sodium level ≥140 mEq/L correlated with significantly improved OS (p=0.04). Patients with sodium levels ≥140 mEq/L after treatment start as well as those with sodium levels ≥140 mEq/L at both pre- and post-ICI evaluation had longer PFS (p=0.05 and p<0.01, respectively) and OS (p<0.01) compared to patients with sodium levels <140 mEq/L. Notably, patients with sodium levels ≥140 mEq/L at the post-ICI evaluation and at both pre- and post-ICI evaluation displayed a better DCR (p=0.01 and p<0.01, respectively). These results align with our recent study, which demonstrated that lower sodium levels (<140 mEq/L) were associated with worse PFS and OS in mRCC patients receiving TKIs as first-line or nivolumab as second-line therapy (20, 21). It is important to note that higher baseline sodium levels do not significantly impact PFS in these patients. This may be due to the unique mechanisms of immunotherapy, including delayed response, atypical tumor responses (*e.g.*, pseudo-progression), and establishment of long-lasting immune memory, may explain why improvements in OS are observed without immediate impacts on PFS. These factors underscore the complexity of assessing treatment efficacy and emphasize the importance of considering long-term benefits in outcome assessments.

Serum sodium levels are routinely measured but not clearly defined for prognostic proposes in mRCC. Nonetheless, previous evidence has linked hyponatremia with poor prognosis in various cancers, including RCC (14–16, 33, 34). Notably, Rinaldi et al., evaluated the prognostic impact of hyponatremia in NSCLC patients with BMs. They found that patients with BMs and hyponatremia had a mOS of 10.1 months versus 13.1 months for eunatremic patients with BMs, suggesting an important prognostic role of sodium level in the management these patients (35). Several causes of cancer-associated hyponatremia have been proposed (36–39), but the pathophysiology of hyponatremia is not yet fully understood.

Recent hypotheses suggest that upregulated sodium-transporting proteins my contribute to intracellular sodium accumulation in cancer cells, promoting invasiveness and poorer prognosis (22). Additionally, emerging evidence implicates sodium storage in immune system modulation, potentially influencing cancer treatment outcomes (23). In particular, the intake of sodium may potentially affect the activation state of the immune system by directly impacting T helper cell subtypes and innate immune cells in various tissues (23). Furthermore, it has been shown that an elevated consumption of sodium can alter the makeup of the intestinal microbiota, resulting in indirect impacts on immune cells (23). These discoveries suggest that sodium might have regulatory functions in several health conditions, including cardiovascular disease, inflammation, infection, autoimmunity, and could potentially even be relevant in the context of cancer treatment.

A recent *post hoc* analysis of the IMmotion151 and IMvigor 211 phase 3 clinical trials indicated that elevated baseline sodium levels are associated with a positive response to immunotherapy and improved outcomes in patients with mRCC and metastatic urothelial carcinoma receiving immunotherapy (40). Unlike other key serum electrolytes such as potassium, magnesium, and calcium, sodium alone shows a linear correlation with favorable prognosis during immunotherapy, suggesting a potentially beneficial role for increased sodium levels. Importantly, after adjusting for prognostic factors, elevated sodium levels did not improve prognosis in the comparator arms of the trials, which involved sunitinib and chemotherapy, respectively. This implies that the predictive value of baseline sodium may be specific to immunotherapy (40).

This study, in line with our previous findings (20, 21), underscores a potential correlation between elevated sodium levels and improved response to ICI in patients with RCC and BMs. These findings have the potential to enhance the management of mRCC patients. Overall, we can hypothesize that patients at low risk of developing hyponatremia before ICI treatment have a stronger likelihood of improved outcomes. Additionally, patients who maintain normal serum sodium levels after exposure to ICI tend to responde better. This raises the possibility of integrating serum sodium levels into patient risk assessments and may serve as an impetus to involve consultants, such as nephrologists, earlier, who can focus on managing natremia.

This research has various limitations, primarily due to its retrospective nature and the utilization of second-line therapy, which is no longer the standard of care, except in specific scenarios. Furthermore, the comprehensive assessment of patient comorbidities and their concurrent medications, particularly antihypertensive drugs, was omitted. Moreover, numerous factors, such as the circumstances at the time of sampling or prior nephrectomy, have the potential to impact the sodium levels in these individuals (37).

Notwithstanding these constraints, the study boasts several strengths, including participation from multiple healthcare centers, the inclusion of a substantial patient cohort, and the examination of natremia at both baseline and post-treatment initiation. Moreover, we recognized that further validation in an independent cohort could be necessary to confirm the utility of this biomarker in these patients. These findings will also be investigated in the ongoing prospective study Meet-URO 33 (41).

## Conclusion

In summary, our investigation has unveiled that among RCC patients with BMs treated with nivolumab as a second-line therapy or beyond, the presence of a pre-ICI serum sodium level of ≥140 mEq/L is associated with extended OS compared to those with sodium levels <140 mEq/L. Furthermore, individuals who have sodium levels ≥140 mEq/L after starting treatment demonstrate superior PFS, OS, and DCR in contrast to those with levels <140 mEq/L. Additionally, patients consistently exhibiting sodium values ≥140 mEq/L both before and after ICI administration consistently display enhanced OS, PFS, and DCR relative to patients with sodium levels ≥140 mEq/L at either time point. Higher sodium levels may constitute a crucial factor linked to improved survival outcomes in RCC patients with BMs undergoing immunotherapy, implying its potential inclusion as an additional parameter in patients’ risk assessments. Further investigations are warranted to validate our findings.

## Data availability statement

The raw data supporting the conclusions of this article will be made available by the authors, without undue reservation.

## Ethics statement

The studies involving humans were approved by Ethics Regional Ethical Committee of Liguria, under registration number 068/2019. The studies were conducted in accordance with the local legislation and institutional requirements. The human samples used in this study were acquired from primarily isolated as part of your previous study for which ethical approval was obtained. Written informed consent for participation was not required from the participants or the participants’ legal guardians/next of kin in accordance with the national legislation and institutional requirements. Written informed consent was obtained from the individual(s) for the publication of any potentially identifiable images or data included in this article.

## Author contributions

MaC: Conceptualization, Data curation, Formal analysis, Writing – original draft, Writing – review & editing. SR: Conceptualization, Data curation, Writing – original draft, Writing – review & editing. MM: Data curation, Resources, Writing – original draft, Writing – review & editing. UG: Investigation, Writing – original draft, Writing – review & editing. SB: Supervision, Visualization, Writing – original draft, Writing – review & editing. LG: Methodology, Resources, Writing – original draft, Writing – review & editing. GF: Data curation, Writing – original draft, Writing – review & editing. PZ: Resources, Supervision, Writing – original draft, Writing – review & editing. MeC: Writing – original draft, Writing – review & editing. SC: Resources, Writing – original draft, Writing – review & editing. IZ: Writing – original draft, Writing – review & editing. SP: Writing – original draft, Writing – review & editing. RR: Data curation, Writing – original draft, Writing – review & editing. MS: Validation, Writing – original draft, Writing – review & editing, Supervision. VM: Investigation, Resources, Writing – original draft, Writing – review & editing. MT: Resources, Writing – original draft, Writing – review & editing. LF: Writing – original draft, Writing – review & editing, Resources, Supervision. VP: Resources, Writing – review & editing. OC: Resources, Validation, Writing – review & editing. FA: Conceptualization, Writing – review & editing. FM: Resources, Writing – review & editing. GP: Supervision, Writing – review & editing. FN: Resources, Supervision, Writing – review & editing. FV: Resources, Writing – review & editing. AC: Resources, Writing – original draft. MN: Resources, Writing – review & editing. AM: Resources, Writing – review & editing. EN: Supervision, Validation, Writing – review & editing. AS: Resources, Writing – review & editing. GB: Supervision, Validation, Writing – review & editing. PR: Resources, Validation, Writing – review & editing. LC: Supervision, Validation, Writing – review & editing. LA: Supervision, Validation, Writing – review & editing. GR: Conceptualization, Data curation, Formal analysis, Methodology, Software, Supervision, Validation, Writing – original draft, Writing – review & editing.
