# Supplementary material for: Sodium levels and immunotherapy efficacy in mRCC patients with bone metastases: sub analysis of Meet-Uro 15 study
Source: Front Immunol. 2024 Jul 5;15:1361010. doi: 10.3389/fimmu.2024.1361010 (PMC11257879; doi:10.3389/fimmu.2024.1361010)
Supplement: Supplementary file 1 [file Table_1.docx]

**Table S1.** Patients’ baseline characteristics according to pre-treatment Na+.

|  | ≥140 mEq/L  69 (57.5%) | <140 mEq/L  51 (42.5%) | *p* |
| --- | --- | --- | --- |
| Age  Median (range) | 75 (45-84) | 76 (44-83) | 0.9 |
| Gender, n (%)  Male | 50 (72.5) | 37 (72.5) | 0.9 |
| Histology, n (%)  Clear-cell RCC | 64 (92.7) | 44 (88) | 0.5 |
| Previous nephrectomy n (%)  Yes | 58 (84.1) | 40 (78.4) | 0.7 |
| Karnofsky performance status, n (%)  ≥80% | 56 (81.2) | 39 (76.5) | 0.6 |
| IMDC score, n (%)  Intermediate-poor | 61 (88.4) | 43 (84.3) | 0.6 |
| Sites of metastases, n (%)  Lymph-nodal  Visceral | 38 (55.1)  57 (82.6) | 29 (56.9)  43 (84.3) | 0.8  0.9 |
| First-Line Therapy, n (%)  Sunitinib  Pazopanib | 44 (65.7)  23 (34.3) | 29 (58)  21 (42) | 0.4 |
| Nivolumab line, n (%)  Second line  ⩾Third line | 46 (66.7)  23 (33.3) | 39 (76.5)  12 (23.5) | 0.3 |

*RCC: renal cell carcinoma; IMDC:* [*international metastatic renal cell carcinoma database consortium*](https://www.imdconline.com/)*; TKI: tyrosine kinase inhibitor.*

**Table S2.** Patients’ baseline characteristics according to first evaluation Na+.

|  | ≥140 mEq/L  56 (46.6%) | <140 mEq/L  64 (53.3%) | *p* |
| --- | --- | --- | --- |
| Age  Median (range) | 76 (44-80) | 76 (45-84) | 0.9 |
| Gender, n (%)  Male | 44 (78.6) | 43 (67.2) | 0.2 |
| Histology, n (%)  Clear-cell RCC | 51 (91.1) | 57 (90.1) | 0.9 |
| Previous nephrectomy n (%)  Yes | 50 (89.3) | 48 (75.0) | 0.1 |
| Karnofsky performance status, n (%)  ≥80% | 43 (76.8) | 52 (81.2) | 0.6 |
| IMDC score, n (%)  Intermediate-poor | 48 (85.7) | 56 (87.5) | 0.8 |
| Sites of metastases, n (%)  Lymph-nodal  Visceral | 33 (58.9)  46 (82.1) | 34 (53.1)  54 (84.4) | 0.6  0.8 |
| First-Line Therapy, n (%)  Sunitinib  Pazopanib | 34 (62.7)  20 (37.0) | 39 (61.9)  24 (24.1) | 0.9 |
| Nivolumab line, n (%)  Second line  ⩾Third line | 38 (67.9)  18 (32.1) | 47 (73.4)  17 (27.6) | 0.5 |

*RCC: renal cell carcinoma; IMDC:* [*international metastatic renal cell carcinoma database consortium*](https://www.imdconline.com/)*; TKI: tyrosine kinase inhibitor.*

**Table S3.** Patients’ baseline characteristics according to pre-and first assessment Na+.

|  | ≥140 mEq/L  41 (34.1%) | <140 mEq/L  79 (58.3%) | *p* |
| --- | --- | --- | --- |
| Age  Median (range) | 76 (54-80) | 76 (44-84) | 0.9 |
| Gender, n (%)  Male | 32 (78.0) | 55 (69.9) | 0.4 |
| Histology, n (%)  Clear-cell RCC | 37 (90.2) | 71 (91.0) | 0.9 |
| Previous nephrectomy n (%)  Yes | 37 (90.2) | 61 (77.2) | 0.1 |
| Karnofsky performance status, n (%)  ≥80% | 34 (82.9) | 61 (77.2) | 0.6 |
| IMDC score, n (%)  Intermediate-poor | 34 (82.9) | 70 (88.6) | 0.4 |
| Sites of metastases, n (%)  Lymph-nodal  Visceral | 24 (58.5)  33 (80.5) | 43 (54.4)  67 (84.8) | 0.7  0.6 |
| First-Line Therapy, n (%)  Sunitinib  Pazopanib | 25 (62.5)  15 (37.5) | 48 (62.3)  29 (37.7) | 0.9 |
| Nivolumab line, n (%)  Second line  ⩾Third line | 26 (63.4)  15 (36.6) | 59 (74.7)  20 (25.3) | 0.2 |

*RCC: renal cell carcinoma; IMDC:* [*international metastatic renal cell carcinoma database consortium*](https://www.imdconline.com/)*; TKI: tyrosine kinase inhibitor.*
